# Burden and trends of antimicrobial non-susceptibility in skin and soft tissue infections: nine-year microbiological surveillance from a tertiary hospital in Riyadh, Saudi Arabia

**DOI:** 10.3389/fmicb.2025.1712297

**Published:** 2026-01-09

**Authors:** Yahya Shabi, Abdullah A. Alshehri, Khalifa Binkhamis, Mohammed Alqahtani, Thamir Saad Alsaeed, Ali Abdullah Aljaberi, Saleh Abdullah Alkhamis, Mohammad K. Alshomrani, Abdullah Z. Almutairi, Abdulah J. Alqahtani, Ahmad Jebril M. Bosaily, Fatimah Alshahrani

**Affiliations:** 1 Department of Microbiology and Clinical Parasitology, College of Medicine, King Khalid University, Abha, Saudi Arabia; 2 Microbiology Laboratory, Prince Mohammed bin Abdulaziz Hospital, Riyadh, Saudi Arabia; 3 Department of Pathology, College of Medicine, King Saud University, Riyadh, Saudi Arabia; 4 Microbiology Section, Pathology and Medical Laboratories, Department and Blood Banks, Security Forces Hospital, Riyadh, Saudi Arabia; 5 Department of Biology and Immunology, College of Medicine, Qassim University, Qassim, Saudi Arabia; 6 Microbiology Department, Riyadh Regional Laboratory, Riyadh, Saudi Arabia; 7 Microbiology Laboratory, King Fahad Hospital, Medina, Saudi Arabia; 8 Department of Surgery, College of Medicine, King Khalid University, Abha, Saudi Arabia; 9 Division of Infectious Diseases, Department of Internal Medicine, King Saud University Medical City, King Saud University, Riyadh, Saudi Arabia; 10 College of Medicine, King Saud University, Riyadh, Saudi Arabia

**Keywords:** antimicrobial resistance, carbapenem-resistant Enterobacterales, *Escherichia coli*, methicillin-resistant *Staphylococcus aureus*, multidrug resistance, non-susceptibility, *Pseudomonas aeruginosa*, Saudi Arabia

## Abstract

**Background:**

Skin and soft tissue infections (SSTIs) impose a substantial global and regional burden, and their management is increasingly complicated by antimicrobial non-susceptibility. In Saudi Arabia, data remain fragmented, with few studies providing species-level analyses stratified by specimen type and infection depth.

**Methods:**

We retrospectively analyzed 6,760 wound and tissue specimens (2016–2024) from a tertiary hospital in Riyadh, Saudi Arabia. Organisms were identified using standard microbiological methods and VITEK 2. Antimicrobial susceptibility testing was interpreted according to CLSI M100, defining non-susceptibility as resistant or intermediate categories. Binary logistic regression was used to assess temporal trends in antimicrobial non-susceptibility, with year of isolation entered as a continuous predictor.

**Results:**

Gram-negative organisms predominated (63.2%), followed by Gram-positives (35.6%) and yeast (1.2%). *Staphylococcus aureus* was the leading pathogen (28.8%), with methicillin resistance detected in 39.0% of isolates. *Escherichia coli* (14.7%) and *Pseudomonas aeruginosa* (12.1%) were also common. Among Enterobacterales, 26.9% were extended-spectrum *β*-lactamase (ESBL) producers and 16.1% were carbapenem-resistant Enterobacterales (CRE). *P. aeruginosa* showed high carbapenem non-susceptibility. Tissue-derived isolates demonstrated significantly higher meropenem non-susceptibility than swab Isolates (20.3% vs. 16.4%, *p* = 0.027), although Enterobacterales subsets occasionally showed the reverse pattern. Temporal analysis revealed rising non-susceptibility to amikacin, ceftriaxone, imipenem, and meropenem (*p* < 0.05), while oxacillin resistance in *S. aureus* and clindamycin non-susceptibility in Gram-positives declined over time.

**Conclusion:**

Gram-negative organisms predominated in SSTIs, showing rising non-susceptibility to amikacin and carbapenems. Separately, among Gram-positive pathogens, *S. aureus* exhibited a clear decline in oxacillin resistance. These shifts underscore the need for ongoing resistance surveillance.

## Introduction

Skin and soft tissue infections (SSTIs) encompass a wide clinical spectrum, ranging from mild cellulitis and abscesses to severe necrotizing infections involving fascia and muscle compartments. They represent a significant global health burden and carry considerable economic implications, as resistant or complicated cases are associated with prolonged hospital stays, higher healthcare costs, and increased morbidity and mortality (Stevens et al., 2014). Antimicrobial resistance (AMR) further complicates management by limiting therapeutic options and increasing the risk of treatment failure and recurrence.

Recent global assessments indicate that the burden of SSTIs continues to rise, particularly in regions with expanding antimicrobial resistance (AMR). Large-scale analyses highlight increasing contributions from priority pathogens including *Escherichia coli*, *Klebsiella pneumoniae*, *Pseudomonas aeruginosa*, and *Acinetobacter baumannii* to SSTI morbidity, driven by multidrug resistance and the spread of high-risk clones (Gajic et al., 2025; Gillet et al., 2023). In the Middle East, AMR pressure is further amplified by regional population mobility and mass gatherings, which have been linked to increased carriage of ESBL-producing Enterobacterales and other resistant organisms (Lwigale et al., 2024). National data similarly show a growing impact of carbapenem-resistant non-fermenters and extended-spectrum β-lactamase (ESBL)-producing Enterobacterales on SSTIs, underscoring the need for updated, species-level surveillance to guide empirical therapy in Saudi Arabia (Deplano et al., 2023; Lwigale et al., 2024). Moreover, the rising economic and healthcare burden associated with AMR highlights the urgency of local epidemiological studies that can inform stewardship efforts and optimize antimicrobial use (Teo et al., 2025).

Worldwide, *S. aureus* remains the most common cause of wound and tissue infections, with methicillin-resistant strains (MRSA) constituting a persistent therapeutic challenge. MRSA prevalence varies widely across regions, reflecting differences in antimicrobial use and infection control practices, but in many countries exceeds 30% of *S. aureus* isolates. Gram-negative bacilli, including *E. coli*, *K. pneumoniae*, and *P. aeruginosa*, have also emerged as major contributors to SSTIs, particularly in chronic wounds, surgical site infections, and healthcare-associated cases, where multidrug non-susceptibility is increasingly observed (Khalid et al., 2024; Peetermans et al., 2020; Stevens et al., 2014).

In the Middle East, *S. aureus* remains a leading cause of wound infections. In Saudi Arabia, MRSA prevalence among *S. aureus* wound isolates has been reported at 37–40%, although methicillin-susceptible strains remain slightly more frequent (Almutairi et al., 2024; Binsuwaidan et al., 2023; Khalid et al., 2024). Gram-negative bacilli such as *E. coli*, *K. pneumoniae*, and *P. aeruginosa* also contribute substantially to wound and tissue infections, particularly in chronic and healthcare-associated cases (Khalid et al., 2024; Peetermans et al., 2020). Alarming non-susceptibility patterns have been documented across the country. Extended-spectrum β-lactamase (ESBL) production and carbapenem non-susceptibility are increasingly reported among Enterobacterales wound isolates (Khalid et al., 2024). In Makkah, surgical wound infections demonstrated very high multidrug resistance (MDR) rates, with *Acinetobacter baumannii* (97%), *K. pneumoniae* (81%), *E. coli* (71%), and MRSA (60%) predominating, along with vancomycin-resistant *enterococci* (22%) (6). Similarly, long-term surveillance in eastern Saudi Arabia showed that the proportion of community-acquired MRSA among all MRSA isolates increased from 20 to 59% over an eight-year period (Almutairi et al., 2024). Beyond MRSA, Gram-negative bacilli pose significant challenges. In Najran, 84% of clinical isolates were MDR, 10% were extensively drug-resistant (XDR), and 6% were pandrug-resistant (PDR), with *E. coli*, *S. aureus*, *K. pneumoniae*, and *A. baumannii* most commonly implicated (Almakrami et al., 2024). A decade-long analysis of *P. aeruginosa* clinical isolates in Saudi Arabia revealed carbapenem non-susceptibility in 40%, MDR in 37.5%, XDR in 5.3%, and difficult-to-treat resistance (DTR) in 3.5% (Shabi et al., 2025).

Despite multiple regional reports, comprehensive species-level analyses stratified by specimen type and wound depth remain limited. Addressing this gap is critical for guiding empirical therapy and stewardship Therefore, this study aimed to describe the microbiological spectrum of wound and tissue infections in Saudi Arabia, evaluate antimicrobial non-susceptibility patterns and assess temporal trends in non-susceptibility over a nine-year period, with further subgroup analysis by specimen type and infection depth.

## Materials and methods

### Study design and setting

This retrospective cross-sectional study was conducted at Prince Mohammed bin Abdulaziz Hospital, a tertiary care center in Riyadh, Saudi Arabia. It included all patients with skin and soft tissue infections (SSTIs) from January 2016 to December 2024. Microbiological and antimicrobial susceptibility data were collected from laboratory records to assess pathogen distribution, non-susceptibility patterns by specimen type, and temporal trends in non-susceptibility.

### Sample collection and microbiological processing

Clinical specimens including wound and abscess swabs, purulent aspirates, and tissue biopsies were collected from patients with suspected SSTIs and processed according to standard microbiological protocols. Direct Gram staining was followed by inoculation onto 5% sheep blood agar (Oxoid, United Kingdom), MacConkey agar (Oxoid, United Kingdom), and chocolate agar (bioMérieux, France) incubated aerobically with 5% CO₂ at 35–37 °C. Anaerobic cultures were performed on 5% sheep blood agar incubated at 35–37 °C. Robertson’s Cooked Meat (RCM) broth (Oxoid, United Kingdom) incubated aerobically at 35–37 °C. Cultures were examined daily for 48 h. extended up to 5 days for deep site specimens (e.g., tissue, aspirates). Anaerobic plates were read at 48 h. and RCM broth was Gram-stained and subcultured if turbidity developed. All media underwent quality control upon receipt according to CLSI M22 and M100 standards. Bacterial identification was based on colony morphology, Gram stain, and biochemical tests. Species-level confirmation using the VITEK 2 system (bioMérieux, France). In polymicrobial cultures, the dominant pathogen was defined by semiquantitative growth, smear findings, and relevant clinical information. Antimicrobial susceptibility testing (AST) was performed on the VITEK 2 system using appropriate panels (AST-P580, P586, P576, N291, N292, N417, N419, YS07, YS08), supplemented when indicated by disk diffusion on Mueller–Hinton agar (Oxoid, United Kingdom) or broth microdilution in Mueller–Hinton broth for selected agents (e.g., colistin). For all manual AST methods, the turbidity of bacterial suspensions was adjusted to a 0.5 McFarland standard (approximately 1.5 × 10^8^ CFU/mL), in accordance with CLSI M100 recommendations. Testing was organism specific, and not all antimicrobials were applied to every isolate. The panels collectively covered β-lactams, aminoglycosides, fluoroquinolones, macrolides/lincosamides, tetracyclines, trimethoprim–sulfamethoxazole, rifampicin, glycopeptides, and antifungals (fluconazole, voriconazole, caspofungin, micafungin, amphotericin B).

### Data collection and management

Microbiological and clinical data were retrieved from the institutional laboratory information system. Only unique clinical isolates were included, defined as the first isolate per patient per infection episode. Duplicate entries and *Aeromonas* spp. were excluded due to distinct non-susceptibility and ecological profiles. Data extracted included patient demographics, specimen type (e.g., swab and biopsy), organism identification, and AST results. Organisms were grouped as Gram-positive or Gram-negative based on standard classification.

### Statistical analysis

Statistical analyses were conducted using IBM SPSS Statistics, Version 26.0 (IBM Corp., Armonk, NY, United States). Descriptive statistics were used to summarize the dataset. Categorical variables were reported as frequencies and percentages, while continuous variables were reported as medians with interquartile ranges (IQR).

For each antimicrobial agent, susceptibility results were re-coded into a binary categorical variable (susceptible vs. non-susceptible, with intermediate grouped under non-susceptible) according to CLSI M100, Associations between categorical variables were analyzed using Chi-square or Fisher’s exact tests, depending on cell counts. The Chi-square test of independence was used to evaluate associations between bacterial groups or genus (e.g., *Enterococcus*, *Staphylococcus*, *Streptococcus*) and susceptibility status (susceptible vs. non-susceptible) for specific antimicrobial agents. When expected cell counts were <5 or for 2 × 2 Contingency Table Fisher’s exact test was applied instead.

The Mann–Whitney *U* test was used to compare patients age between clinical settings (inpatient vs. outpatient) and Kruskal–Wallis was used to compare patients age between pathogens groups, as age data were not normally distributed. Antimicrobial susceptibility testing (AST) results were examined by cross-tabulation to compare susceptibility distributions between organism types and specimen sources (e.g., tissue vs. swab). Stratification was performed by organism group (Gram-positive or Gram-negative).

For analyses comparing non-susceptibility proportions between organism groups, Fisher’s exact test with risk analysis was applied to calculate odds ratios (ORs) and 95% confidence intervals (CIs).

Temporal trends in non-susceptibility (2016–2024) were assessed using binary logistic regression, which is suitable for dichotomous outcomes (non-susceptible vs. susceptible). The dependent variable was defined as non-susceptibility (coded as 1 = resistant or intermediate, 0 = susceptible), and the independent variable was the year of isolation, which is appropriately treated as a continuous predictor in temporal trend analysis following established international AMR surveillance methodologies (European Centre for Disease Prevention and Control [ECDC], 2023; World Health Organization, 2022).

To ensure model stability, the year variable (2016–2024) was median-centered by subtracting the median study year (2019) from each observation (i.e., centered year = year − 2019). After centering, the value of 0 corresponds to the year 2019, which therefore becomes the implicit reference point for the model intercept. Centering does not alter the odds ratio or statistical significance; it simply rescales the predictor and improves interpretability of the regression coefficients. The odds ratio (OR) thus represents the multiplicative change in the odds of non-susceptibility for each one-year increase relative to this centered baseline (2019). An OR greater than 1 indicates an increasing annual probability of non-susceptibility, whereas an OR less than 1 reflects a decreasing trend. Models were stratified by organism groups based on antimicrobial spectrum.

This approach estimates the annual change in odds of non-susceptibility and enables computation of the annual percent change (APC) from the odds ratio, using the formula:

APC = (Exp(B) − 1) × 100,

where Exp(B) represents the exponentiated logistic regression coefficient.

Regression outputs included the *p-value*, odds ratio [Exp(B)], and 95% confidence interval (CI).

Binary logistic regression was chosen because it provides a quantitative effect size for year-to-year change and aligns with trend-analysis approaches recommended by global AMR surveillance systems such as WHO GLASS and EARS-Net (European Centre for Disease Prevention and Control [ECDC], 2023; World Health Organization, 2022). Temporal changes were also illustrated using line graphs to support the regression findings, a two-tailed *p*-value < 0.05 was considered statistically significant.

A line graph, generated using Python (version 3.11), was used to visualize the annual percentage of non-susceptible isolates for key pathogens, complementing the regression findings.

In all analyses, non-susceptibility was defined to include both resistant and intermediate categories in accordance with CLSI M100 guidelines. This terminology was applied consistently throughout the study to enhance comparability.

## Results

### Distribution of demographic and clinical characteristics by organism category

A total of 6,760 patients with wound or tissue cultures were included in the study. The majority were hospitalized 80.1% (5,416/6760), while the remaining 19.9% (1,344/6760) were treated in outpatient settings. The overall median (interquartile range, IQ) of patient ages was 54.0 (31.0) years. Inpatients were significantly older than outpatients [56.0 (30.0) vs. 46.0 (29.0) years; *p* < 0.001]. Male patients comprised 61.7% (4,172/6760) of the study and were more likely to be hospitalized than females (81.0% vs. 78.7%, *p* = 0.028).

Gram-negative bacteria were the predominant pathogens, isolated in 63.2% (4,273/6760) of cases, followed by Gram-positive bacteria (2,405/6760, 35.6%) and yeast (82/6760, 1.2%). Patient age also differed significantly by pathogen type (Kruskal–Wallis *H* = 45.908, df = 2, *p* < 0.001): those with Gram-negative infections were older on average (56.18 ± 19.39 years) than those with Gram-positive (52.91 ± 19.35 years) or yeast infections (51.29 ± 15.44 years).

Pathogen distribution varied by clinical setting. Of the 6,760 isolates, most were obtained from inpatients 80.1% (5,416/6760), while 19.9% (1,344/6760) came from outpatients. Among inpatients, the majority of Isolates were from non-ICU wards (4,817/5416, 88.9%), with the remaining 11.1% (599/5416) from ICU patients. In non-ICU wards, Gram-negative organisms accounted for 66.0% (3,179/4817) of isolates, Gram-positive organisms for 32.8% (1,578/4817), and yeast for 1.2% (60/4817). In ICU Isolates, Gram-negative organisms were even more predominant 79.0% (473/599), compared with 18.7% (122/599) Gram-positive and 2.3% (14/599) yeast.

Among outpatients (*n* = 1,344), Gram-positive organisms were slightly more frequent (715/1344, 53.2%) than Gram-negative (621/1344, 46.2%), while yeast accounted for 0.6% (8/1344). Most outpatient Isolates were obtained from the emergency department (1,277/1344, 95.0%), where 52.6% (672/1277) of isolates were Gram-positive, 46.8% (598/1277), were Gram-negative, and only 0.6% (7/1277) were yeast. Clinic Isolates (67/1344, 5.0%) showed a similar pattern, with (43/67, 64.2%) Gram-positive, (23/67, 34.3%) Gram-negative, and (1/67, 1.5%) yeast. Overall, Gram-negative organisms predominated in inpatient, particularly ICU, settings, whereas Gram-positive organisms were slightly more common among outpatient, emergency department, and clinic isolates.

Body-site distribution also varied across pathogen categories. Gram-negative organisms predominated in trunk (2,159/3058, 70.6%) and perineal infections (196/256, 76.6%), whereas Gram-positive organisms were relatively more frequent in head and neck (43.3%) and extremity infections (1,305/3023, 43.2%). Yeast isolates were uncommon across all body sites, ranging from1.0% (30/3023) to 2.4% (10/423) (Table 1).

**Table 1:** Distribution of demographic and clinical characteristics according to isolated organism categories in tissue and wounds cases.

Characteristic	Total isolates (*n* = 6,760)	Gram negative (*n* = 4,273)	Gram positive (*n* = 2,405)	Yeast (*n* = 82)
Age [Median (IQR)]	54.0 (31.0)	56.0 (31.0)	52.0 (29.0)	51.0 (19.25)
Sex				
Male	4,172 (61.7%)	2,619 (62.8%)	1,502 (36.0%)	51 (1.2%)
Female	2,588 (38.3%)	1,654 (63.9%)	903 (34.9%)	31 (1.2%)
In-patient	5,416 (80.1%)	3,652 (67.4%)	1,690 (31.2%)	74 (1.4%)
Non-ICU Ward	4,817 (88.9%)	3,179 (66.0%)	1,578 (32.8%)	60 (1.2%)
ICU Ward	599 (11.1%)	473 (79.0%)	122 (18.7%)	14 (2.3%)
Out-patient	1,344 (19.9%)	621 (46.2%)	715 (53.2%)	8 (0.6%)
ER	1,277 (95.0%)	598 (46.8%)	672 (52.6%)	7 (0.6%)
Clinic	67 (5.0%)	23 (34.3%)	43 (64.2%)	1 (1.5%)
Sample type				
Swab	5,410 (80.0%)	3,464 (64.0%)	1909 (35.3%)	37 (0.7%)
Tissue	1,350 (20.0%)	809 (59.9%)	496 (36.7%)	45 (3.3%)
Body site				
Head and neck	423 (6.3%)	230 (54.4%)	183 (43.3%)	10 (2.4%)
Trunk	3,058 (45.2%)	2,159 (70.6%)	860 (28.1%)	39 (1.3%)
Extremities	3,023 (44.7%)	1,688 (55.8%)	1,305 (43.2%)	30 (1.0%)
Perineum	256 (3.8%)	196 (76.6%)	57 (22.3%)	3 (1.2%)

ICU, intensive care unit; ER, emergency room; IQR, Interquartile range. Of the total 6,760 isolates, Gram-negative organisms constituted 63.2% (*n* = 4,273), Gram-positive 35.6% (*n* = 2,405), and yeast 1.2% (*n* = 82).

### Distribution of swab and tissue isolates by anatomical site

**Figure 1:**
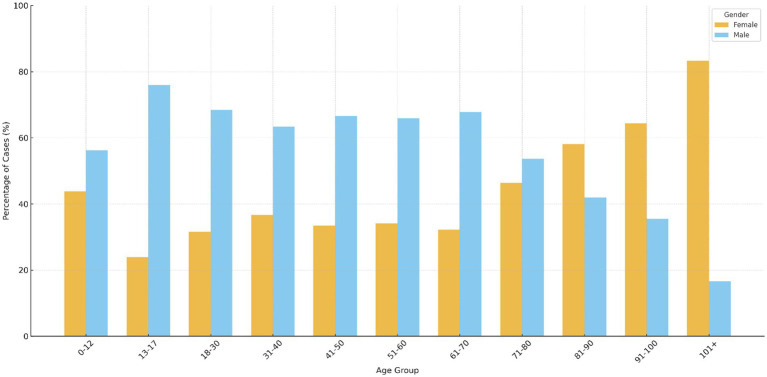
Percentage distribution of wound and tissue infection cases across age groups stratified by gender. Clustered bars represent the proportion of male and female patients within each age group. Values are expressed as percentages of the total number of isolates within each age category. The figure displays the proportional distribution of male and female isolates across age groups, highlighting the relative gender composition within each age category rather than absolute isolate counts.

A total of 6,760 wound and tissue Isolates were analyzed, comprising 80.0% (5,410/6760) swabs and 20.0% (1,350/6760) tissue specimens. The distribution of sample types varied significantly across body sites (*p* < 0.001). Extremities showed the highest proportion of tissue Isolates (868/3023, 28.7%), whereas head and neck (50/423, 11.8%), trunk (395/3058, 12.9%), and perineum (37/256, 14.5%) sites yielded lower proportions of tissue specimens. Conversely, swab Isolates predominated across all anatomical regions, notably in head and neck (373/423, 88.2%), trunk (2,663/3058, 87.1%), and perineum (219/256, 85.5%) sites. Tissue specimens represented the smallest proportion of Isolates (1,350/6760, 20.0%) (Supplementary Table S1) (Figure 1).

### Distribution of bacterial isolates by gram stain and taxonomic group

Of the 6,678 *bacterial* isolates analyzed representing all Gram-positive and Gram-negative organisms identified in this study and excluding the 82 yeast isolates reported separately in Table 1 Gram-negative bacteria accounted for 64.0% (4,273/6678), and Gram-positive bacteria for 36.0% (2,405/6678). Within the Gram-negative group, Enterobacterales were predominant, comprising 70.0% (2,993/4273) of Gram-negative isolates and 44.8% (2,993/6678) of the total bacterial population. Non-fermenting Gram-negative bacilli represented the remaining (1,280/4273, 30.0%), accounting for 19.2% (1,280/6678) of total isolates. Among Gram-positive organisms, *Staphylococcus* species were the most frequent (1981/2405, 82.4%) of Gram-positive isolates and (1981/6678, 29.7%) of all bacterial isolates, followed by *Enterococcus* (325/2405, 13.5%) and *Streptococcus* species (99/2405, 4.1%) (Table 2).

**Table 2:** Distribution of bacterial isolates by Gram stain classification and taxonomic grouping.

Gram type	Organism category	Count	% of total bacterial isolates (*n* = 6,678*)	% within gram type
Gram-Negative	Non-fermenter	1,280	19.2%	30.0%
	Enterobacterales	2,993	44.8%	70.0%
	Total	4,273	64.0%	100.0%
Gram-Positive	*Enterococcus*	325	4.8%	13.5%
	*Staphylococcus*	1981	29.7%	82.4%
	*Streptococcus*	99	1.5%	4.1%
	Total	2,405	36.0%	100.0%

*Totals in this table (*n* = 6,678) represent bacterial isolates only. The difference from the overall isolate count in Table 1 (*n* = 6,760) corresponds to the 82 yeast isolates excluded from this bacterial subanalysis.

### Predominant bacterial species isolated from wound and tissue specimens

*Staphylococcus aureus* was the most frequently isolated species, accounting for 28.8% (1926/6678). Of these, 61.0% (1,175/1926) were methicillin-susceptible (MSSA) and 39.0% (751/1926) were methicillin-resistant (MRSA). *Escherichia coli* was the second most common pathogen (992/6678, 14.7%), followed by *P. aeruginosa* (818/6678, 12.2%) and *K. pneumoniae* (841/6678, 12.6%). Together, *S. aureus*, *E. coli*, and *P. aeruginosa* accounted for 55.9% (3,736/6678) of all bacterial isolates.

The distribution of these predominant species varied by specimen type. In superficial wound isolates, *S. aureus* remained the leading pathogen (934/6678, 13.8%), followed by *P. aeruginosa* (447/6678, 6.7%), *K. pneumoniae* (446/6678, 6.7%), and *E. coli* (423/6678, 6.3%). In deep wound isolates, *S. aureus* was again the most common species (687/6678, 10.2%), followed by *E. coli* (408/6678, 6.1%), *K. pneumoniae* (241/6678, 3.6%), and *P. aeruginosa* (230/6678, 3.4%). In tissue specimens, *S. aureus* also predominated (305/6678, 4.5%), followed by *E. coli* (161/6678, 2.4%), *K. pneumoniae* (154/6678, 2.3%), and *P. aeruginosa* (141/6678, 2.1%) (Supplementary Table S2).

### Antimicrobial non-susceptible profile

#### Comparative non-susceptibility profiles between non-fermenters and Enterobacterales

Among the 4,273 Gram-negative isolates, antimicrobial non-susceptibility varied substantially between non-fermenting Gram-negative bacilli (*n* = 1,280) and Enterobacterales (*n* = 2,993). When considering only isolations with valid AST results for each antibiotic, non-fermenters consistently exhibited markedly higher non-susceptibility to several key agents. These differences were most pronounced for piperacillin–tazobactam (92/250, 36.8%) in non-fermenters vs. (97/548, 17.7%) in Enterobacterales, cefepime (393/1105, 35.6%) vs. (769/2597, 29.6%), Ceftriaxone (68/80, 85.0%) vs. (937/2252, 41.6%), imipenem (365/965, 37.8%) vs. (440/2190, 20.1%), meropenem (337/951, 35.4%) vs. (221/2301, 9.6%), and sulfamethoxazole–trimethoprim (394/433, 91.0%) vs. (65/87, 74.7%). All these differences were statistically significant (*p* < 0.001, Fisher’s exact test).

In contrast, differences in non-susceptibility observed between non-fermenters and Enterobacterales for amikacin (106/912, 11.6%) vs. (314/2858, 11.0%), gentamicin (257/1038, 24.8%) vs. (536/2375, 22.6%), tobramycin (144/755, 19.1%) vs. (43/176, 24.4%), ceftazidime (400/1091, 36.7%) vs. (954/2520, 37.9%), ciprofloxacin (383/1069, 35.8%) vs. (871/2453, 35.5%), and levofloxacin (282/861, 32.8%) vs. (71/255, 27.8%) were not significant (*p* > 0.05) (Table 3).

**Table 3:** Comparison of the profile of non-fermenters and Enterobacterales associated with wound and tissue infections against Gram-negative-targeted antibiotics.

Antibiotic	Total isolates	Gram-negative		*P-v*alue	OR (95%CI)
		Non-fermenter	Enterobacterales		
Amikacin^b^	420/3,770 (11.1%)	106/912 (11.6%)	314/2,858 (11.0%)	0.587^a^	0.939 (0.743–1.186)
Gentamicin^c^	793/3,413 (23.2%)	257/1,038 (24.8%)	536/2,375 (22.6%)	0.172^a^	0.886 (0.747–1.051)
Tobramycin	187/931 (20.1%)	144/755 (19.1%)	43/176 (24.4%)	0.117^a^	1.372 (0.930–2.024)
Piperacillin-Tazobactam	189/798 (23.7%)	92/250 (36.8%)	97/548 (17.7%)	**<0.001** ^**a**^	0.369 (0.263–0.518)
Ceftazidime	1,354/3,611 (37.5%)	400/1,091 (36.7%)	954/2,520 (37.9%)	0.501^a^	1.052 (0.908–1.219)
Cefepime	1,162/3,702 (31.4%)	393/1,105 (35.6%)	769/2,597 (29.6%)	**<0.001** ^**a**^	0.762 (0.656–0.885)
Ceftriaxone	1,005/2,332 (43.1%)	68/80 (85.0%) ^d^	937/2,252 (41.6%)	**<0.001** ^**a**^	0.126 (0.068–0.234)
Imipenem	805/3,155 (25.5%)	365/965 (37.8%)	440/2,190 (20.1%)	**<0.001** ^**a**^	0.413 (0.350–0.488)
Meropenem	558/3,252 (17.2%)	337/951 (35.4%)	221/2,301 (9.6%)	**<0.001** ^**a**^	0.194 (0.160–0.235)
Ciprofloxacin	1,254/3,522 (35.6%)	383/1,069 (35.8%)	871/2,453 (35.5%)	0.878^a^	0.986 (0.849–1.146)
Levofloxacin	353/1,116 (31.6%)	282/861 (32.8%)	71/255 (27.8%)	0.146^a^	0.792 (0.582–1.079)
Sulfamethoxazole-Trimethoprim	459/520 (88.3%)	394/433 (91.0%) ^e^	65/87 (74.7%)	**<0.001** ^**a**^	0.292 (0.163–0.525)

^a^Fisher’s exact test; percentages represent the proportion of isolates classified as non-susceptible (intermediate + resistant) according to CLSI M100; not all Gram-positives isolates were tested against every agent; ^b^Amikacin breakpoints for *Pseudomonas aeruginosa* are designated for urine isolates only as per CLSI M100-Ed33 (2023); ^c^Gentamicin breakpoints for *Pseudomonas aeruginosa* were officially removed by CLSI in the 2023 update (M100-Ed33); ^d^non-fermenter excludes *Pseudomonas aeruginosa* and Stenotrophomonas; ^e^non-fermenter excludes *Pseudomonas aeruginosa*. Percentages represent the proportion of isolates classified as non-susceptible (intermediate + resistant) according to CLSI M100. Bold values indicate statistical significance at *p* < 0.05.

### Non-susceptibility patterns among Gram-positive genera

Among the 2,405 Gram-positive isolates, substantial and statistically significant variation in non-susceptibility was observed across *Enterococcus* (*n* = 325), *Staphylococcus* (*n* = 1,981), and *Streptococcus* (*n* = 99). Fluoroquinolone non-susceptibility was highest in *Enterococcus*, with (106/278, 38.1%) non-susceptible to levofloxacin and (22/72, 30.6%) to moxifloxacin. These rates were lower in *Staphylococcus* (447/1611, 27.7%) for levofloxacin and (362/1531, 23.6%) for moxifloxacin and lowest in *Streptococcus* (4/88, 4.5%) and (4/85, 4.7%), respectively, with *p* < 0.001 for both comparisons.

Clindamycin resistance demonstrated the most pronounced genus-level disparity: *Streptococcus* showed extremely high non-susceptibility (60/63, 95.2%), compared with only (287/1853, 15.5%) in *Staphylococcus* (*p* < 0.001). *Enterococcus* was classified as intrinsically resistant and therefore appropriately excluded from testing.

Tetracycline non-susceptibility followed a similar pattern, being highest in *Streptococcus* (71/86, 82.6%) and *Enterococcus* (173/255, 67.8%), with substantially lower rates in *Staphylococcus* (197/1383, 14.2%) (*p* < 0.001).

Vancomycin non-susceptibility was detected exclusively in *Enterococcus* (18/240, 7.5%), with all *Staphylococcus* (0/1241, 0.0%) and *Streptococcus* (0/83, 0.0%) isolates fully susceptible. Ampicillin resistance likewise occurred only in *Enterococcus* (60/316, 19.0%), while all *Streptococcus* isolates (88/88, 100.0%) remained susceptible; ampicillin is not tested for *Staphylococcus*. While Oxacillin non-susceptibility used exclusively to determine methicillin susceptibility among *Staphylococcus* species, showed non-susceptibility in (776/1970, 39.4%) isolates.

Non-susceptibility to sulfamethoxazole–trimethoprim was not common overall and noted only in *Enterococcus* (9/84, 10.7%), with no resistance observed in *Streptococcus* (0/15, 0.0%). This difference was not statistically significant (*p* = 0.347) (Table 4).

**Table 4:** Comparative non-susceptibility rates to Gram-positive-targeted antibiotics among *Enterococcus*, *Staphylococcus*, and *Streptococcus* isolates in wound and tissue infections.

Antibiotic	Total isolates	Gram-positive			*P-*value
		*Enterococcus*	*Staphylococcus*	*Streptococcus*	
Levofloxacin	557/1977 (28.2%)	106/278 (38.1%)	447/1,611 (27.7%)	4/88 (4.5%)	**<0.001** ^**a**^
Moxifloxacin	388/1,688 (23.0%)	22/72 (30.6%)	362/1,531 (23.6%)	4/85 (4.7%)	**<0.001** ^**a**^
Clindamycin	392/1961 (20.0%)	45/45 (100%)	287/1853 (15.5%)	60/63 (95.2%)	**<0.001** ^**a**^
Ampicillin	60/404 (14.9%)	60/316 (19.0%)	—	0/88 (0.0%)	-
Oxacillin^b^	776/1970 (39.4%)	—	776/1970 (39.4%)	—	-
Tetracycline	441/1724 (25.6%)	173/255 (67.8%)	197/1,383 (14.2%)	71/86 (82.6%)	**<0.001** ^**a**^
Vancomycin	18/1,564 (1.2%)	18/240 (7.5%)	0/1,241 (0.0%)	0/83 (0.0%)	-
Sulfamethoxazole-Trimethoprim	9/99 (9.1%)	9/84 (10.7%)	—	0/15 (0.0%)	-

^a^Chi square test; ^b^oxacillin is used for *Staphylococcus* organisms only; not all Gram-positive isolates were tested against every agent, non-susceptibility include (intermediate and non-susceptible). Percentages represent the proportion of isolates classified as non-susceptible (intermediate + resistant) according to CLSI M100. Bold values indicate statistical significance at *p* < 0.05.

### Temporal trends in antimicrobial non-susceptibility

Logistic regression analysis revealed several significant year-to-year changes in antimicrobial non-susceptibility among wound and tissue isolates between 2016 and 2024. For Gram-negative organisms, increasing trends were observed for amikacin (odds ratio [OR] = 1.060; 95% CI: 1.018–1.104; *p* = 0.004), corresponding to an annual percent change (APC) of +6.0%, and for the carbapenems imipenem (OR = 1.073; 95% CI: 1.039–1.108; *p* < 0.001; APC + 7.3%) and meropenem (OR = 1.073; 95% CI: 1.034–1.112; *p* < 0.001; APC + 7.3%). A similar upward trend was seen for ceftriaxone (OR = 1.060; 95% CI: 1.018–1.104; *p* = 0.004; APC + 6.0%).

Conversely, declining non-susceptibility trends were detected for cefepime (OR = 0.954; 95% CI: 0.928–0.981; *p* = 0.001; APC − 4.6%) and gentamicin (OR = 0.955; 95% CI: 0.925–0.987; *p* = 0.005; APC − 4.5%). Among Gram-positive organisms, oxacillin non-susceptibility in *S. aureus* decreased significantly (OR = 0.956; 95% CI: 0.921–0.993; *p* = 0.019; APC − 4.4%), and clindamycin non-susceptibility across Gram-positives showed a marked downward trajectory (OR = 0.924; 95% CI: 0.881–0.968; *p* = 0.001; APC − 7.6%). In the combined dataset, levofloxacin non-susceptibility also declined over time (OR = 0.957; 95% CI: 0.926–0.989; *p* = 0.008; APC − 4.3%).

**Figure 2:**
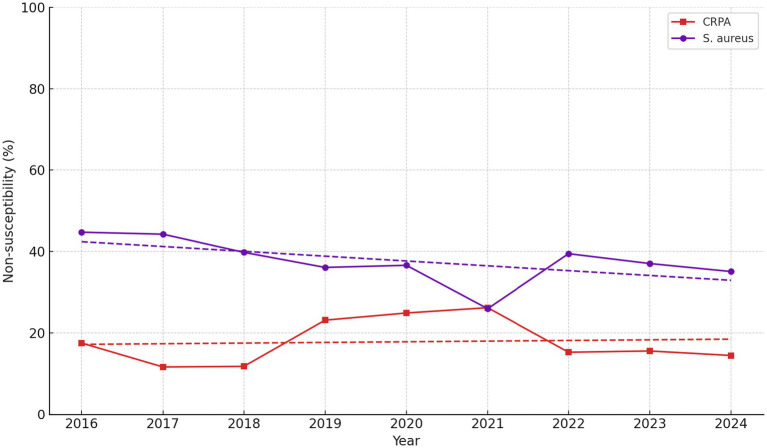
Temporal non-susceptibility trends in *Pseudomonas aeruginosa* and *Staphylococcus aureus* isolated from wounds and tissues between 2016 and 2024. The red line represents carbapenem non-susceptibility in *P. aeruginosa* (CRPA), while the purple line indicates oxacillin non-susceptibility in *S. aureus*. Trend lines are based on linear regression to highlight temporal patterns. CRPA rates remained relatively stable with interannual variation, whereas *S. aureus* demonstrated a sustained decline in oxacillin non-susceptibility. Non-susceptibility includes resistant and intermediate categories, defined according to CLSI M100 guidelines. Values are shown as annual percentages of non-susceptible isolates.

No statistically significant changes were identified for ciprofloxacin, piperacillin-tazobactam, tobramycin, tetracycline, vancomycin, or trimethoprim–sulfamethoxazole (*p* > 0.05). These temporal patterns for *S. aureus* and *P. aeruginosa* (CRPA) are further illustrated in Figure 2, which depicts the annual percentages of carbapenem-resistant *P. aeruginosa* (CRPA) and oxacillin-resistant *S. aureus* across the study period. The figure shows that CRPA rates fluctuate between years without a consistent upward or downward trend, whereas oxacillin resistance in *S. aureus* demonstrates a gradual decline over time, partially consistent with the direction and magnitude of trends presented in Table 5.

**Table 5:** Logistic regression models for antibiotics non-susceptibility trends among Gram-negative, Gram-positive, and combined species in tissue and wound infections.

Family	Antimicrobial	Susceptibility profile	EXP(B)	95% CI	*p*-value^a^	APC
Gram-negative	Amikacin	Non-susceptible (Reference)	1	—	—	—
		Susceptible	**1.060**	1.018–1.104	**0.004**	**+6.0%**
	Ceftazidime	Non-susceptible (Reference)	1	—	—	—
		Susceptible	0.974	0.948–1.001	0.058	NS
	Cefepime	Non-susceptible (Reference)	1	—	—	—
		Susceptible	**0.954**	0.928–0.981	**0.001**	**−4.6%**
	Ciprofloxacin	Non-susceptible (Reference)	1	—	—	—
		Susceptible	0.991	0.964–1.019	0.543	NS
	Gentamicin	Non-susceptible (Reference)	1	—	—	—
		Susceptible	**0.955**	0.925–0.987	**0.005**	**−4.5%**
	Imipenem	Non-susceptible (Reference)	1	—	—	—
		Susceptible	**1.073**	1.039–1.108	**<0.001**	**+7.3%**
	Meropenem	Non-susceptible (Reference)	1	—	—	—
		Susceptible	**1.073**	1.034–1.112	**<0.001**	**+7.3%**
	Piperacillin-tazobactam	Non-susceptible (Reference)	1	—	—	—
		Susceptible	1.069	0.881–1.298	0.498	NS
	Tobramycin	Non-susceptible (Reference)	1	—	—	—
		Susceptible	0.991	0.924–1.063	0.801	NS
Gram-positive	Clindamycin	Non-susceptible (Reference)	1	—	—	—
		Susceptible	**0.924**	0.881–0.968	**0.001**	**−7.6%**
	Moxifloxacin	Non-susceptible (Reference)	1	—	—	—
		Susceptible	1.001	0.956–1.049	0.956	NS
	Oxacillin^b^	Non-susceptible (Reference)	1	—	—	—
		Susceptible	**0.956**	0.921–0.993	**0.019**	**−4.4%**
	Tetracycline	Non-susceptible (Reference)	1	—	—	—
		Susceptible	1.042	0.998–1.089	0.064	NS
	Vancomycin	Non-susceptible (Reference)	1	—	—	—
		Susceptible	1.189	0.989–1.429	0.066	NS
Combined	Levofloxacin	Non-susceptible (Reference)	1	—	—	—
		Susceptible	**0.957**	0.926–0.989	**0.008**	**−4.3%**
	Sulfamethoxazole-Trimethoprim	Non-susceptible (Reference)	1	—	—	—
		Susceptible	1.019	0.909–1.143	0.748	NS

^a^Year is entered as predictor variable for each antibiotic. ^b^Oxacillin is applicable to *Staphylococcus aureus* isolates. APC (annual percent change) was calculated as (OR−1) × 100(OR − 1) × 100(OR−1) × 100. “NS” = not significant (*p* > 0.05). Bold values indicate statistical significance at *p* < 0.05.

### Distribution of non-susceptibility and species subgroups

Subgroup analysis revealed a substantial burden of non-susceptibility among key bacterial genera. Within the Enterobacterales group (*n* = 2,993), ESBL strains comprised 26.9% (805/2993) and carbapenem-resistant Enterobacterales (CRE) accounted for 16.1% (482/2993) of Enterobacterales, highlighting a high prevalence of multidrug resistance. Among Staphylococcus isolates (*n* = 1,981), methicillin-resistant *S. aureus* (MRSA) represented 39.0% (751/1981) of the Enterobacterales, while methicillin-susceptible *S. aureus* (MSSA) was more common (1,175/1981, 61.0%) and coagulase-negative *staphylococci* (CoNS) constituted a minor proportion of the isolates (55/1981, 2.8%). In the *Enterococcus* group (*n* = 325), *E. faecalis* was predominant (249/325, 76.6%), followed by *E. faecium* (63/325, 19.4%) and other species (13/325, 4.0%).

### Subgroup analysis by sample type and wound depth

Stratified analysis revealed notable variations in non-susceptibility rates among Gram-negative isolates based on sample type and wound depth. As shown in Supplementary Table S3, non-susceptible patterns between swab and tissue Isolates were generally comparable, though meropenem non-susceptibility was significantly higher in tissue-derived isolates (123/606, 20.3%) vs. (435/2646, 16.4%), *p* = 0.027. For non-fermenting Gram-negative bacilli (Supplementary Table S4), no statistically significant differences were observed between Gram-negative isolates from superficial and deep wound across most antibiotics, including imipenem and meropenem.

Further analysis focused on tissue specimens specifically (Supplementary Table S5), where non-fermenters exhibited high non-susceptibility rates to ceftazidime (75/189, 39.7%), imipenem (67/168, 39.9%), and piperacillin-tazobactam (34/76, 44.7%). Among Enterobacterales (Supplementary Table S6), deep wound isolates showed significantly lower non-susceptibility rates to key agents such as amikacin (175/1324, 13.2%) vs. (76/987, 7.7%), *p* < 0.001, cefepime (397/1208, 32.9%) vs. (227/893, 25.4%), *p* < 0.001, and meropenem (123/1075, 11.4%) vs. (39/784, 5.0%), *p* < 0.001, suggesting higher non-susceptibility in superficial wounds. However, ampicillin non-susceptibility remained high in both groups.

In tissue-specific Enterobacterales isolates (Supplementary Table S7), non-susceptibility was highest against ampicillin (292/343, 85.1%), ceftriaxone (196/402, 48.8%), and ceftazidime (194/476, 40.8%), reflecting a considerable burden of *β*-lactam non-susceptibility in deep-seated infections.

### Gram-positive non-susceptibility patterns stratified by sample type

Non-susceptibility rates for Gram-positive isolates were stratified by sample type (Supplementary Table S8). Tetracycline non-susceptibility was significantly higher in tissue isolates (137/379, 36.1%) compared with swab isolates (304/1345, 22.6%) (*p* < 0.001). Vancomycin non-susceptibility was also higher in tissue specimens (8/349, 2.3%) than in swabs (10/1215, 0.8%) (*p* = 0.040). Clindamycin non-susceptibility showed moderately higher rates in tissue isolates (82/351, 23.4%) compared with swabs isolates (310/1610, 19.3%), although the difference was not statistically significant (*p* = 0.090), while oxacillin non-susceptibility of isolates from swabs and tissues was followed a similar pattern, with (142/325, 43.7%) in tissue isolates versus (635/1646, 38.6%) in swabs, respectively. The difference in all cases was not significant (*p* = 0.093). Non-susceptibility rates for moxifloxacin, levofloxacin, ampicillin, and sulfamethoxazole-trimethoprim were similar across both sample types with no statistically significant differences observed.

### Antifungal susceptibility patterns in yeast isolates

Among yeast isolates recovered from wounds and tissues, high susceptibility was observed across all tested antifungal agents. Micafungin and flucytosine demonstrated complete susceptibility (100%). Voriconazole also exhibited excellent activity, with only one non-susceptible isolation (1.4%). Fluconazole and caspofungin showed high susceptibility of 97.1 and 92.3%, respectively. Notably, non-susceptibility remained low among yeast isolates. Fluconazole showed non-susceptibility in 2 isolates, while caspofungin demonstrated non-susceptibility in 6 isolates. No non-susceptibility was detected for micafungin, flucytosine, or voriconazole.

## Discussion

This analysis characterized the microbiological spectrum of wound and tissue infections, evaluated antimicrobial non-susceptibility profiles, and examined temporal and specimen-specific variations in non-susceptibility of isolates included in the study. Consistent with global and regional findings, Gram-negative organisms predominated in our study, surveillance showing the growing role of Enterobacterales and non-fermenters as major drivers of complicated SSTIs, including surgical site infections, burn wounds, and diabetic foot ulcers (Binsuwaidan et al., 2023; Khalid et al., 2024; Peetermans et al., 2020).

Recent analyses further indicate that rising AMR pressure particularly in regions such as the Middle East has intensified the clinical impact of MDR *E. coli*, *K. pneumoniae*, and *A. baumannii* in wound infections (Gajic et al., 2025; Gillet et al., 2023; Lwigale et al., 2024). Additionally, population mobility associated with mass gatherings in Saudi Arabia has been linked to increased carriage of ESBL-producing Enterobacterales, reinforcing the relevance of our findings within the national epidemiological context (Deplano et al., 2023; Lwigale et al., 2024).

*Staphylococcus aureus* remained the leading Gram-positive pathogen, with MRSA accounting for 39.0% of *S. aureus* isolates in our study. This proportion is comparable to recent Saudi surveillance studies, where MRSA prevalence ranged from 37 to 40% (Almutairi et al., 2024; Binsuwaidan et al., 2023; Khalid et al., 2024). Importantly, regional data indicate that the proportion of community-acquired MRSA among all MRSA isolates increased from 20 to 59% in eastern Saudi Arabia over an eight-year period (Almutairi et al., 2024). These findings underscore the persistent burden of MRSA in both community and hospital settings and highlight the need for vigilance in empirical therapy choices, as inappropriate initial coverage is associated with worse outcomes in SSTIs.

The downward trend in oxacillin non-susceptibility observed in our *S. aureus* isolates is consistent with recent molecular epidemiology studies reporting stabilization or modest declines in MRSA resistance, alongside the emergence of evolving MSSA lineages with distinct resistance patterns (Lwigale et al., 2024). These shifts highlight the need to monitor both MRSA and MSSA as active causative agent to SSTIs.

Non-fermenting Gram-negative bacilli demonstrated significantly higher non-susceptibility rates compared with Enterobacterales, particularly against piperacillin–tazobactam, third-generation cephalosporins, and carbapenems. This is consistent with recent Saudi studies highlighting multidrug-resistant (MDR) *Pseudomonas* and *Acinetobacter* as major therapeutic obstacles in wound and surgical site infections (Almakrami et al., 2024; Al-Said et al., 2023; Shabi et al., 2025). In our data, non-susceptibility against carbapenem, was especially notable among *P. aeruginosa*, reflecting a broader regional pattern in which 40% of isolates showed carbapenem non-susceptibility, 37.5% were MDR, and 5.3% exhibited XDR phenotypes (Shabi et al., 2025). Similar trends have been reported globally, where non-susceptibility in non-fermenters is strongly associated with prolonged hospitalization, prior carbapenem exposure, and ICU stays, raising concern about limited therapeutic options and increased healthcare costs.

Enterobacterales in this study also displayed concerning non-susceptibility levels, with ESBL production observed at 26.9% and carbapenem non-susceptibility in 16.1% of isolates. These findings are consistent with multicenter Saudi reports showing increasing rates of ESBL-producing and carbapenem-resistant strains in wound infections (Khalid et al., 2024). Such levels of non-susceptibility mirror regional surveillance from the Gulf and align with international reports where ESBL-producing Enterobacterales are increasingly identified in both hospital- and community-onset wound infections. This trend poses a direct challenge for empirical therapy, as standard regimens (such as ceftriaxone or piperacillin–tazobactam) may be rendered ineffective, necessitating early carbapenem or combination therapy in selected high-risk cases. These high non-susceptibility rates suggest that empiric use of third-generation cephalosporins may not be effectively efficacious in high-risk wound infections, necessitating reconsideration of treatment algorithms.

Our findings are consistent with global surveillance patterns. According to the WHO Global Antimicrobial Resistance Surveillance System (GLASS), median non-susceptibility rates across 76 countries reached 42% for third-generation cephalosporin–resistant *E. coli* and 35% for MRSA (World Health Organization, 2022). A systematic review of AMR in the Middle East further demonstrated median carbapenem non-susceptibility of 74.2% in *Acinetobacter* spp., with ESBL prevalence of 32% in *E. coli* and 28% in *K. pneumoniae*, and carbapenem non-susceptibility of 8.1 and 15.4%, respectively (Truppa and Abo-Shehada, 2020). In Europe, the EARS-Net 2023 report documented a 57.5% increase in carbapenem-resistant *K. pneumoniae* bloodstream infections compared with 2019, while MRSA bloodstream infections decreased by 17.6% (European Centre for Disease Prevention and Control [ECDC], 2023). Locally, a Saudi multicenter surveillance study (2011–2016) showed significant increases in carbapenem non-susceptibility among *P. aeruginosa*, with imipenem and meropenem non-susceptibility increasing by 12.3 and 11.6%, respectively (Somily et al., 2018). More recently, a 10-year review emphasized that non-susceptibility rates across Saudi Arabia are increasing across both Gram-negative and Gram-positive organisms, highlighting the urgent need for national surveillance aligned with GLASS (Thabit et al., 2023).

In addition, our temporal analysis offers important insights into evolving non-susceptibility patterns. We observed annual percent change (APC) in non-susceptibility to amikacin (+6.0% per year) and carbapenems (+7.3% per year for both imipenem and meropenem) among Gram-negative organisms. This rising trajectory is concerning, as these agents often serve as last-line therapies in severe SSTIs, suggesting escalating selective pressure and possible clonal dissemination of resistant strains. Similarly, ceftriaxone non-susceptibility increased significantly (+6.0% per year), reflecting declining utility of third-generation cephalosporins as empiric options in high-risk wound infections. Conversely, significant downward trends were noted for cefepime (−4.6% per year), gentamicin (−4.5% per year), oxacillin in *S. aureus* (−4.4% per year), and clindamycin in Gram-positives (−7.6% per year). These reductions may reflect evolving prescribing practices, shifts in empirical therapy guided by stewardship interventions, or reduced fitness of resistant strains in certain contexts. Notably, declining oxacillin and clindamycin non-susceptibility suggests partial stabilization of MRSA epidemiology in our setting, in contrast to the persistent upward trajectory of Gram-negative carbapenem non-susceptibility.

Increasing non-susceptibility to amikacin, carbapenems, and ceftriaxone mirrors global trends among priority Gram-negative pathogens (Gajic et al., 2025). These shifts have significant clinical and economic implications, as AMR is associated with longer hospital stays, use of broader-spectrum therapies, and increased healthcare costs (Teo et al., 2025). This reinforces the need to strengthen stewardship efforts and optimize empirical choices in severe SSTIs.

Another important observation in our study was the influence of specimen type and infection depth on the likelihood of antimicrobial non-susceptibility among isolates. Tissue-derived isolates exhibited higher non-susceptibility to agents such as meropenem and tetracycline compared to superficial swab specimens. This may reflect greater antimicrobial exposure, biofilm formation, and selective pressure in deep or chronic infections. Previous Saudi reports from Makkah similarly documented very high MDR rates in surgical wounds, particularly among *A. baumannii* (97%) and *K. pneumoniae* (81%) (Al-Said et al., 2023). These findings highlight the importance of specimen type in interpreting microbiological data, as superficial swabs may underestimate the burden of non-susceptibility in deeper tissues.

The high burden of Gram-negative organisms with broad-spectrum non-susceptibility particularly to carbapenems and extended-spectrum cephalosporins, is particularly concerned given the limited therapeutic options available. Although our dataset was not stratified by MDR/XDR/PDR categories, such none-susceptibility profiles have been reported regionally. For example, in Najran, 84% of isolates were MDR, 10% XDR, and 6% PDR (Almakrami et al., 2024). The emergence of difficult-to-treat resistant (DTR) *P. aeruginosa* (Shabi et al., 2025) and vancomycin-resistant Gram-positive pathogens (Al-Said et al., 2023) further exemplifies the narrowing therapeutic landscape. Regional studies have reported high MDR and XDR burdens among *E. coli*, *K. pneumoniae*, and *A. baumannii* in wound and surgical-site infections across the Gulf region (Deplano et al., 2023; Lwigale et al., 2024). The elevated non-susceptibility rates observed in our isolates, particularly among Gram-negative pathogens are consistent with broader regional resistance pressures. Together, these observations highlight the urgent need for comprehensive national surveillance programs, integration of molecular non-susceptibility data, and targeted stewardship interventions to curb the spread of high-risk clones and non-susceptibility determinants. Our findings reinforce the urgency of strengthening antimicrobial stewardship programs and implementing standardized surveillance reporting across Saudi Arabia.

This study benefits from a long surveillance period (2016–2024) and a large number of wound and tissue specimens, enabling robust organism-level and temporal analyses. Stratification by specimen type and infection depth provided clinically relevant insights that are often absent from routine surveillance datasets. However, several limitations should be noted. As a single-center retrospective study, generalizability may be limited, and the absence of detailed clinical data prevented assessment of patient-level risk factors or outcomes. Molecular confirmation of resistance mechanisms was not performed, and reliance on superficial swabs in routine practice may underestimate resistance in deep infections. Fungal isolates were infrequent, limiting interpretation of antifungal susceptibility patterns.

## Conclusion

This nine-year analysis highlights the microbiological profile and antimicrobial non-susceptibility patterns of wound and tissue infections in Saudi Arabia. Gram-negative organisms accounted for most isolates, with particularly elevated non-susceptibility among non-fermenters. Enterobacterales demonstrated notable ESBL and carbapenem non-susceptibility, while *S. aureus* remained the leading Gram-positive pathogen, with MRSA representing more than one-third of isolates. Temporal analysis showed rising non-susceptibility to key agents such as amikacin, carbapenems, and ceftriaxone, alongside declining trends for oxacillin and clindamycin. Tissue-derived isolates exhibited higher non-susceptibility than superficial swabs, underscoring the clinical importance of specimen depth when interpreting culture results. These findings provide actionable data for empirical therapy selection and highlight the need for continued surveillance and stewardship efforts tailored to local epidemiology.

## Data Availability

The original contributions presented in the study are included in the article/Supplementary material, further inquiries can be directed to the corresponding author.

## References

[ref1] Almakrami M. Salmen M. Aldashel Y. A. Alyami M. H. Alquraishah N. AlZureea M. . (2024). Prevalence of multidrug-, extensively drug-, and pandrug-resistant bacteria in clinical isolates from king Khaled Hospital, Najran, Saudi Arabia. Discover Med. 1:108. doi: 10.1007/s44337-024-00094-8

[ref2] Almutairi H. Alanazi F. Aljohani A. Alghamdi S. Alshahrani A. Alenzi A. . (2024). Rising prevalence of methicillin-resistant *Staphylococcus aureus* in eastern Saudi Arabia: 8-year surveillance. New Microbes New Infect. 59:101152. doi: 10.1016/j.nmni.2024.101152

[ref3] Al-Said H. M. Alghamdi A. Ashgar S. S. Jalal N. A. Faidah H. S. Johargy A. K. . (2023). Isolation and detection of drug-resistant bacterial pathogens in postoperative wound infections at a tertiary care hospital in Saudi Arabia (2017–2021). Saudi J. Med. Med. Sci. 11, 229–234. doi: 10.4103/sjmms.sjmms_405_22, 37533663 PMC10393095

[ref4] Binsuwaidan R. Khan M. A. Alzahrani R. H. Aldusaymani A. M. Almallouhi N. M. Alsabti A. S. . (2023). Prevalence of multidrug-resistant and ESBL-producing bacterial pathogens in patients with chronic wound infections and spinal cord injury admitted to a tertiary care rehabilitation hospital. Antibiotics. 12:1587. doi: 10.3390/antibiotics12111587, 37998789 PMC10668744

[ref5] Deplano A. Hallin M. Bustos Sierra N. Michel C. Prevost B. Martiny D. . (2023). Persistence of the *Staphylococcus aureus* epidemic European fusidic acid-resistant impetigo clone (EEFIC) in Belgium. J. Antimicrob. Chemother. 78, 2061–2065. doi: 10.1093/jac/dkad204, 37358399 PMC10393872

[ref6] European Centre for Disease Prevention and Control [ECDC] (2023). Antimicrobial resistance in the EU/EEA (EARS-net) – Annual epidemiological report 2023. Stockholm: ECDC.

[ref7] Gajic I. Tomic N. Lukovic B. Jovicevic M. Kekic D. Petrovic M. . (2025). A comprehensive overview of antibacterial agents for combating multidrug-resistant bacteria: the current landscape, development, future opportunities, and challenges. Antibiotics. 14:221. doi: 10.3390/antibiotics14030221, 40149033 PMC11939824

[ref8] Gillet Y. Lorrot M. Minodier P. Ouziel A. Haas H. Cohen R. (2023). Antimicrobial treatment of skin and soft tissue infections. Infect. Dis. Now 53:104787. doi: 10.1016/j.idnow.2023.104787, 37734714

[ref9] Khalid F. Poulose C. Farah D. F. M. Mahmood A. Elsheikh A. Khojah O. T. (2024). Prevalence and antimicrobial susceptibility patterns of wound and pus bacterial pathogens at a tertiary care hospital in Central Riyadh, Saudi Arabia. Microbiol. Res. 15, 2015–2034. doi: 10.3390/microbiolres15040135

[ref10] Lwigale F. Kibombo D. Kasango S. D. Tabajjwa D. Atuheire C. Kungu J. . (2024). Prevalence, resistance profiles and factors associated with skin and soft-tissue infections at Jinja regional referral hospital: a retrospective study. PLoS Glob. Public Health 4:e0003582. doi: 10.1371/journal.pgph.0003582, 39093883 PMC11296629

[ref11] Peetermans M. de Prost N. Eckmann C. Norrby-Teglund A. Skrede S. De Waele J. J. (2020). Necrotizing skin and soft-tissue infections in the intensive care unit. Clin Microbiol Infect. 26, 8–17. doi: 10.1016/j.cmi.2019.06.031 31284035

[ref12] Shabi Y. Algarni A. Al Bshabshe A. Alazraqi T. Patriquin G. Bawazeer A. O. . (2025). A decade of antimicrobial resistance trends in *Pseudomonas aeruginosa*: insights from a tertiary care hospital in Saudi Arabia (2013–2022). Front. Microbiol. 16:1617522. doi: 10.3389/fmicb.2025.1617522 40693146 PMC12277317

[ref13] Somily A. M. AlSubaie S. S. BinSaeed A. A. Al-Aska A. I. Alzamil F. A. Shibl A. M. . (2018). Antimicrobial resistance trends of non-fermenter gram-negative bacteria in Saudi Arabia: a multicenter surveillance study, 2011–2016. J. Infect. Public Health 11, 614–619. doi: 10.1016/j.jiph.2018.04.008 34358816

[ref14] Stevens D. L. Bisno A. L. Chambers H. F. Dellinger E. P. Goldstein E. J. Gorbach S. L. . (2014). Practice guidelines for the diagnosis and management of skin and soft tissue infections: 2014 update by the Infectious Diseases Society of America. Clin. Infect. Dis. 59, 147–159. doi: 10.1093/cid/ciu444 24947530

[ref15] Teo M. Z. Y. Loo H. L. Goh B. H. Chuah L. H. (2025). Progress in topical nanoformulations against bacterial skin and soft tissue infections – current trends. Drug Deliv. Transl. Res. 15, 4141–4186. doi: 10.1007/s13346-025-01924-7, 40681833 PMC12508018

[ref16] Thabit A. K. Alabbasi A. Y. Alnezary F. S. Almasoudi I. A. (2023). An overview of antimicrobial resistance in Saudi Arabia (2013–2023) and the need for national surveillance. Microorganisms 11:2086. doi: 10.3390/microorganisms11082086, 37630646 PMC10460018

[ref17] Truppa C. Abo-Shehada M. N. (2020). Antimicrobial resistance among GLASS pathogens in conflict and non-conflict affected settings in the Middle East: a systematic review. BMC Infect. Dis. 20:936. doi: 10.1186/s12879-020-05503-8, 33297983 PMC7724697

[ref18] World Health Organization (2022). Antimicrobial resistance. Fact sheet. Geneva: WHO.

