# Supplementary material for: Burden and trends of antimicrobial non-susceptibility in skin and soft tissue infections: nine-year microbiological surveillance from a tertiary hospital in Riyadh, Saudi Arabia
Source: Front Microbiol. 2026 Jan 9;16:1712297. doi: 10.3389/fmicb.2025.1712297 (PMC12827636; doi:10.3389/fmicb.2025.1712297)
Supplement: Supplementary file 1 [file Data_Sheet_1.pdf]

Supplementary

Table S1. Distribution of Swab and Tissue Isolates by Anatomical Site

Body Site	Swab<br>5410 / 6760 (80.0%)	Tissue<br>1350 / 6760 (20.0%)
Head and neck	373 / 423 (88.2%)	50 / 423 (11.8%)
Trunk	2663 / 3058 (87.1%)	395 / 3058 (12.9%)
Extremities	2155 / 3023 (71.3%)	868 / 3023 (28.7%)
Perineum	219 / 256 (85.5%)	37 / 256 (14.5%)
Note. Pearson's Chi-square = 262.227, df = 3, p < 0.001.		

Table S2. Predominant Bacterial Species and Their Distribution Across Specimen Types

Species	Total bacterial Isolates	Superficial Wounds	Deep Wounds	Tissue Specimens
Staphylococcus aureus (total)	1926 / 6678 (28.8%)	934 / 6678 (13.8%)	687 / 6678 (10.2%)	305 / 6678 (4.5%)
— MSSA	1175 / 1926 (61.0%)	570/1175 (48.5%)	427/1175 (36.3%)	178/1175 (15.1%)
— MRSA	751 / 1926 (39%)	364/751 (48.5%)	260/751 (34.6%)	127/751 (16.9%)
Escherichia coli	992 / 6678 (14.7%)	423 / 6678 (6.3%)	408 / 6678 (6.1%)	161 / 6678 (2.4%)
Pseudomonas aeruginosa	818 / 6678 (12.2%)	447 / 6678 (6.7%)	230 / 6678 (3.4%)	141/6678 (2.1%)
Klebsiella pneumoniae spp.	841 / 6678 (12.6%)	446 / 6678 (6.7%)	241 / 6678 (3.6%)	154 / 6678 (2.3%)
Note. Percentages for species totals and specimen-type counts are calculated using all bacterial isolates (n = 6,678). MSSA and MRSA percentages in subgroup rows are calculated using *S. aureus* total (n = 1,926) or the subgroup denominator where appropriate.				

Table S3. Non-susceptibility rates for antibiotics specific to Gram negatives stratified by sample type

**Antibiotic**	**Total Isolates**	**Sample type**		***P-Value***	**OR (95%CI)**
		Swab	Tissue		
Amikacin	420 / 3770 (11.1%)	328 / 3057 (10.7%)	92 / 713 (12.9%)	0.099[a]	1.23 (0.96 – 1.58)
Ceftazidime	1354 / 3611 (37.5%)	1085 / 2946 (36.8%)	269 / 665 (40.5%)	0.084[a]	1.16 (0.98 – 1.38)
Cefepime	1162 / 3702 (31.4%)	946 / 3012 (31.4%)	216 / 690 (31.3%)	1.000[a]	0.99 (0.83 – 1.19)
Ciprofloxacin	1254 / 3522 (35.6%)	1034 / 2866 (36.1%)	220 / 656 (33.5%)	0.223[a]	0.89 (0.75 – 1.07)
Imipenem	805 / 3155 (25.5%)	637 / 2561 (24.9%)	168 / 594 (28.3%)	0.095[a]	1.19 (0.98 – 1.46)
Meropenem	558 / 3252 (17.2%)	435 / 2646 (16.4%)	123 / 606 (20.3%)	**0.027[a]**	1.29 (1.04 – 1.62)
Note. a - Fisher's exact test; Percentages represent the proportion of isolates classified as non-susceptible (intermediate + resistant) according to CLSI M100, Bold values indicate statistical significance at p < 0.05.					

Table S4. Non-susceptibility rates for antibiotics specific to non-fermenters stratified by wound depth

Antibiotic	Total Isolates	Wound Depth		*P-Value*	OR (95%CI)
		Superficial wound	Deep wound		
Amikacin	77 / 746 (10.3%)	51 / 484 (10.5%)	26 / 262 (9.9%)	0.900[a]	1.07 (0.65 -1.76)
Ceftazidime	325 / 902 (36.0%)	211 / 581 (36.3%)	114 / 321 (35.5%)	0.828[a]	1.03 (0.78 – 1.38)
Cefepime	322 / 911 (35.3%)	210 / 591 (35.5%)	112 / 320 (35.0%)	0.885[a]	1.02 (0.77 – 1.36)
Ciprofloxacin	321 / 886 (36.2%)	210 / 576 (36.5%)	111 / 310 (35.8%)	0.884[a]	1.03 (0.77 – 1.37)
Imipenem	298 / 797 (37.4%)	190 / 518 (36.7%)	108 / 279 (38.7%)	0.592[a]	0.92 (0.68 – 1.24)
Meropenem	273 / 787 (34.7%)	189 / 520 (36.3%)	84 / 267 (31.5%)	0.180[a]	1.244 (0.91 – 1.70)
Piperacillin-Tazobactam	58 / 174 (33.3%)	38 / 115 (33.0%)	20 / 59 (33.9%)	1.000[a]	0.96 (0.49 – 1.87)
Tobramycin	118 / 625 (18.9%)	73 / 406 (18.0%)	45 / 219 (20.5%)	0.454[a]	0.85 (0.56 – 1.28)
Levofloxacin	243 / 723 (33.6%)	164 / 485 (33.8%)	79 / 238 (33.2%)	0.933[a]	1.03 (0.74 -1.43)

Note. a- Fisher's exact test; Percentages represent the proportion of isolates classified as non-susceptible (intermediate + resistant) according to CLSI M100, Bold values indicate statistical significance at $p < 0.05$.

Table S5. Non-susceptibility rates for antibiotics specific to non-fermenters in Tissue specimens

Antibiotics	Tissue
Amikacin	29 / 166 (17.5%)
Ceftazidime	75 / 189 (39.7%)
Cefepime	71 / 194 (36.6%)
Ciprofloxacin	62 /183 (33.9%)
Imipenem	67 / 168 (39.9%)
Meropenem	64 / 164 (39.0%)
Piperacillin-Tazobactam	34 / 76 (44.7%)
Tobramycin	26 /130 (20.0%)
Levofloxacin	39 / 138(28.3%)

Table S6. Non-susceptibility rates for antibiotics specific to *Enterobacterales* between Deep wound and superficial wound

Antibiotic	Total Isolates	Type of wound		*P-Value*	OR (95%CI)
		Superficial wound	Deep wound		
Amikacin	251 / 2311 (10.9%)	175 / 1324 (13.2%)	76 / 987 (7.7%)	**< 0.001**[a]	1.83 (1.38 – 2.42)
Ceftazidime	760 / 2044 (37.2%)	469 / 1187 (39.5%)	291 / 857 (34.0%)	**0.011**[a]	1.27 (1.06 – 1.53)
Cefepime	624 / 2101 (29.7%)	397 / 1208 (32.9%)	227 / 893 (25.4%)	**< 0.001**[a]	1.44 (1.18 – 1.74)
Ciprofloxacin	713 / 1980 (36.0%)	440 / 1145 (38.4%)	273 / 835 (32.7%)	**0.009**[a]	1.28 (1.06 – 1.55)
Gentamicin	429 / 1916 (22.4%)	249 / 1104 (22.6%)	180 / 812 (22.2%)	0.868[a]	1.02 (0.82 – 1.27)
Ertapenem	29 / 299 (9.7%)	22 / 176 (12.5%)	7 / 123 (5.7%)	0.072[a]	2.37 (0.98 – 5.73)
Imipenem	339 / 1764 (19.2%)	231 / 1023 (22.6%)	108 / 741 (14.6%)	**< 0.001**[a]	1.71 (1.33 – 2.20)
Meropenem	162 / 1859 (8.7%)	123 / 1075 (11.4%)	39 / 784 (5.0%)	**< 0.001**[a]	2.47 (1.70 – 3.58)
Piperacillin-Tazobactam	60 / 377 (15.9%)	44 / 224 (19.6%)	16 / 153 (10.5%)	**0.021**[a]	2.09 (1.13 – 3.87)
Tobramycin	32 / 128 (25.0%)	23 / 81 (28.4%)	9 / 47 (19.1%)	0.293[a]	1.67 (0.70 – 4.01)
Ampicillin	1425 / 1698 (83.9%)	817 / 947 (86.3%)	608 / 751 (81.0%)	**0.003**[a]	1.48 (1.14 – 1.92)
Levofloxacin	54 / 178 (30.3%)	37 / 105 (35.2%)	17 / 73 (23.3%)	0.099[a]	1.79 (0.91 -3.52)
Sulfamethoxazole -Trimethoprim	51 / 69 (73.9%)	36 / 46 (78.3%)	15 / 23 (65.2%)	0.260[a]	1.92 (0.63 – 5.81)

Note. a- Fisher Exact test; Percentages represent the proportion of isolates classified as non-susceptible (intermediate + resistant) according to CLSI M100, Bold values indicate statistical significance at $p < 0.05$.

Table S7. Non-susceptibility rates for antibiotics specific to *Enterobacterales* in Tissue specimens

Antibiotics	Tissue
Amikacin	63 / 547 (11.5%)
Amoxicillin-Clavulanic Acid	84 / 180 (46.7%)
Ceftazidime	194 / 476 (40.8%)
Ceftriaxone	196 / 402 (48.8%)
Cefepime	145 / 496 (29.2%)
Ciprofloxacin	158 / 473 (33.4%)
Gentamicin	107 / 459 (23.3%)
Ertapenem	17 / 108 (15.7%)
Imipenem	101 / 426 (23.7%)
Meropenem	59 / 442 (13.3%)
Piperacillin-Tazobactam	37 / 171 (21.6%)
Tobramycin	11 / 48 (22.9%)
Ampicillin	292 / 343 (85.1%)
Levofloxacin	17 / 77 (22.1%)
Sulfamethoxazole-Trimethoprim	14 / 18 (77.8%)

Table S8. Non-susceptibility rates for antibiotics specific to Gram positive stratified by sample type

Antibiotic	Total Isolates	Sample type		*P-Value*	OR (95%CI)
		Swab	Tissue		
Clindamycin	392 / 1,961 (20.0%)	310 / 1,610 (19.3%)	82 / 351 (23.4%)	0.090[a]	1.28 (0.97–1.69)
Moxifloxacin	388 / 1,688 (23.0%)	310 / 1,365 (22.7%)	78 / 323 (24.1%)	0.607[a]	1.08 (0.82–1.44)
Oxacillin[b]	777 / 1,971 (39.4%)	635 / 1,646 (38.6%)	142 / 325 (43.7%)	0.093[a]	1.24 (0.97–1.57)
Tetracycline	441 / 1,724 (25.6%)	304 / 1,345 (22.6%)	137 / 379 (36.1%)	**< 0.001[a]**	1.94 (1.52–2.48)
Vancomycin	18 / 1,564 (1.2%)	10 / 1,215 (0.8%)	8 / 349 (2.3%)	**0.040[a]**	2.83 (1.11–7.22)
Ampicillin	60 / 404 (14.9%)	33 / 243 (13.6%)	27 / 161 (16.8%)	0.394[a]	1.28 (0.74–2.23)
Levofloxacin	557 / 1,977 (28.2%)	433 / 1,559 (27.8%)	124 / 418 (29.7%)	0.463[a]	1.10 (0.87–1.39)
Sulfamethoxazole-Trimethoprim	9 / 99 (9.1%)	5 / 48 (10.4%)	4 / 51 (7.8%)	0.736[a]	0.73 (0.18–2.90)

Note. a- Fisher exact test; b – oxacillin is used for *staphylococcus* organisms only; Percentages represent the proportion of isolates classified as non-susceptible (intermediate + resistant) according to CLSI M100, Bold values indicate statistical significance at $p < 0.05$.
